# Unopposed estrogen and endometrial cancer: association or causation?

**DOI:** 10.3325/cmj.2019.60.389

**Published:** 2019-08

**Authors:** Andrea LaCroix, Velimir Simunic, Slobodan Vukicevic

**Affiliations:** 1 Women’s Health Center of Excellence, Family Medicine and Public Health, University of California, San Diego, La Jolla, CA, USA; 2 Human Reproduction Unit, Polyclinic IVF, Zagreb, Croatia; 3 Laboratory for Mineralized Tissues, Center of Excellence for Reproductive and Regenerative Medicine, University of Zagreb School of Medicine, Zagreb, Croatia; 4 Centre for Translational and Clinical Research, University of Zagreb School of Medicine, Zagreb, Croatia *slobodan.vukicevic@mef.hr*

**To the Editor:** In the article “What Are the Odds You Will Read This Article?” published in the *Croatian Medical Journal* (1), Hrabač and Trkulja use the example of unopposed estrogen therapy and endometrial cancer to demonstrate the concept of causality (without a single reference). The article is rather a simplistic explanation of different measures of association (effect) and how they relate to epidemiological study designs. Any other example could have been used, and the choice appears to be arbitrary. However, Hrabač and Trkulja used the misleading and potentially harmful example of unopposed estrogen as a uterine carcinogen. The strength and causality of this association is really unquestionable at this point since it has been extensively demonstrated that estrogen exposure increases manifold the risk of uterine cancer (2). Estrogen has officially been classified as a carcinogen in the US (3). Therefore, we hope that the readers of the *CMJ* will not misunderstand the article and assume that there is a controversy about the causal link between estrogen and uterine cancer (2,3). The authors should also have been aware that women with an intact uterus, except when hysterectomized, are never treated with estrogen alone, but to reduce the risk of cancer, they are, when indicated, prescribed estrogen and progestins instead (2).

For non-expert audiences a presentation of odds ratios in regard to non-collapsibility and its interpretation that is sometimes similar to risk ratios would have been a much better choice because that is what scientists really care about most of the time. A few added references may help readers to understand the odds ratios in practice (4-6).

There exist innumerable examples of questionable exposure-disease associations that could have been selected to illustrate points about measures of association in epidemiological studies. A statistical sketch based on wrong premises and not on veritable published medical knowledge might mislead the readers rather than explain concepts about association between two binary variables. Finally, to appreciate the association and causation in epidemiological studies we recommend to readers the historical publications by Bradford Hill, for example “The Environment and Disease: Association or Causation?” (7).
